# Outer membrane lipoproteins: late to the party, but the center of attention

**DOI:** 10.1128/jb.00442-24

**Published:** 2024-12-13

**Authors:** Kerrie L. May, Marcin Grabowicz

**Affiliations:** 1 Department of Microbiology & Immunology, Emory University School of Medicine 12239 , Atlanta, Georgia, USA; 2 Antibiotic Resistance Center, Emory University 1371 , Atlanta, Georgia, USA; 3 Division of Infectious Disease, Department of Medicine, Emory University School of Medicine 12239 , Atlanta, Georgia, USA; University of Notre Dame, Notre Dame, Indiana, USA

**Keywords:** Gram-negative, outer membrane, lipoproteins, antibiotics

## Abstract

An outer membrane (OM) is the hallmark feature that is often used to distinguish “Gram-negative” bacteria. Our understanding of how the OM is built rests largely on studies of *Escherichia coli*. In that organism—and seemingly in all species of the Proteobacterial phyla—the essential pathways that assemble the OM each rely on one or more lipoproteins that have been trafficked to the OM. Hence, the lipoprotein trafficking pathway appeared to be foundational for the ability of these bacteria to build their OM. However, such a notion now appears to be misguided. New phylogenetic analyses now show us that lipoprotein trafficking was likely the very last of the essential OM assembly systems to have evolved. The emergence of lipoprotein trafficking must have been a powerful innovation for the ancestors of Proteobacteria, given how it assumed such a central place in OM biogenesis. In this minireview, we broadly discuss the biosynthesis and trafficking of lipoproteins and ponder why the newest OM assembly system (lipoprotein trafficking) has become so key to building the Proteobacterial OM. We examine the diversity among lipoprotein trafficking systems, noting uniting commonalities and highlighting key differences. Current novel antibiotic development is targeted against a small subset of Proteobacterial species that cause severe human diseases; several inhibitors of lipoprotein biosynthesis and OM trafficking have been recently reported that may become new antibiotics. Understanding the diversity in lipoprotein trafficking may yield selective new antibiotics that preferentially kill important human pathogens while sparing species of normal healthy flora.

## INTRODUCTION

The division of the bacterial world into “Gram-negative” or “Gram-positive” classes—reflecting their staining propensity in the classical Gram stain technique—had long been used to stereotype bacteria into one of two cell envelope architectures. Gram-negative bacteria were distinguished by a thin peptidoglycan cell wall and the presence of a second, outer membrane (OM) that is rich with the glycolipid lipopolysaccharide (LPS) (1). Gram-positive bacteria, on the other hand, lacked an OM and produced a thick cell wall (1). This simple staining-based distinction remains useful in many contexts, but the Gram status of a few key species has tinted perceptions of entire phyla as consisting of either OM-producing diderm Gram-negatives or monoderm Gram-positives. In fact, phyla that are widely considered Gram-positive contain species that produce a diderm architecture, complete with an OM and even LPS (2–5). For example, OM producing bacteria are present among the Bacillota (*nee* Firmicutes) phylum that is widely considered “Gram-positive” (2–5). Recent re-rooting of bacterial phylogeny showed that an early branching led to the Terrabacteria and Gracilicutes taxa and within these are species generally considered “Gram-positive” and “Gram-negative,” respectively (6). However, diderm cell envelope structures are commonly found among disparate Terrabacteria (2–5). This fact now supports a model where the last common bacterial ancestor was very likely a diderm cell (3, 4, 6). Today’s diderms have simply retained and adapted this ancestral diderm cell envelope architecture (2, 5). Meanwhile, Terrabacterial monoderms apparently arose through any of several independent instances of relinquishing the OM (2, 3, 5). This insight into the origins of the OM offers the chance to re-appraise how we understand OM biogenesis pathways in diverse contemporary bacterial species.

**Fig 1:**
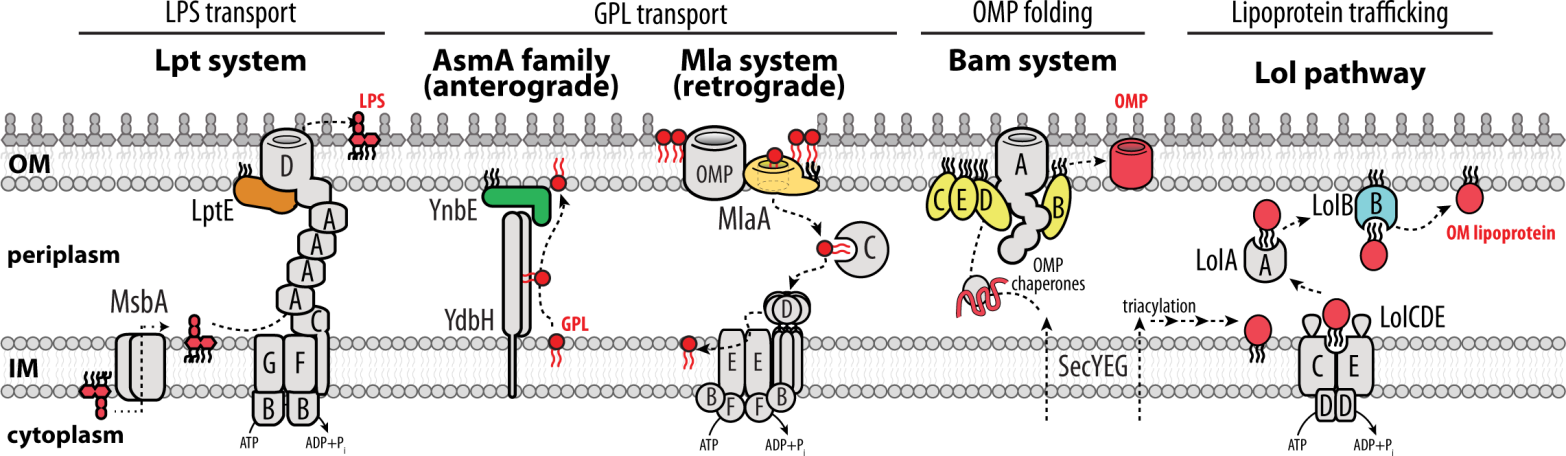
*E. coli* OM assembly pathways rely on OM lipoproteins. OM components assembled by each pathway are red. Lipoproteins of the Lpt, AsmA family, Mla, Bam, and Lol pathways are colored, all other proteins of biogenesis pathways are gray. In wild-type *E. coli*, LptE, BamD, and LolB are each individually essential. The Lpt system relies on LptE to assemble LptD and disaggregate LPS for insertion into the OM outer leaflet. YnbE functions with YdbH to facilitate GPL transport into the OM inner leaflet; note that other, redundant, AsmA-family systems are not currently known to rely on lipoproteins. MlaA is the conduit for return of GPLs that mislocalize in the OM outer leaflet. BamBCDE regulate OMP folding by BamA. LolB receives lipoprotein cargo from LolA and anchors it in the OM inner leaflet. Components of each system are widely conserved among Gracilicutes and Terrabacteria, except for Lol pathway and each of the colored OM lipoproteins which are only well conserved among Gracilicutes.

Our molecular understanding of the OM relies on studies of a narrow set of species, especially the γ-Proteobacteria *Escherichia coli* and *Pseudomonas aeruginosa* and the β-Proteobacteria *Neisseria meningitidis* (1, 7, 8). The OM has proved to be an essential organelle in all diderm species tested—making the repeated loss of the OM by monoderms even more remarkable. The OM is a complex structure of lipids and membrane proteins assembled within an aqueous periplasmic compartment that lacks ready access to chemical energy sources (e.g. ATP) (Fig. 1) (9). *E. coli* studies show that the OM is built as a bilayer with asymmetrically distributed lipids (8, 10, 11). The glycolipid LPS forms the surface-exposed outer leaflet (9). The chemical composition of LPS varies considerably even with species, but all are built on a lipid A precursor molecule (for simplicity, we will refer to all lipid A glycolipids as “LPS”) (12–14). Once synthesized in the cytoplasmic membrane (CM), LPS is translocated across the CM bilayer to the periplasmic leaflet and is then ready for transport to the OM outer leaflet via the LPS transport (Lpt) transenvelope bridge, consisting of seven essential proteins (Fig. 1). The OM inner leaflet consists of glycerophospholipids (GPLs) (9, 15). Current models suggest that proteins from the AsmA protein family (of which at least three are redundant in *E. coli*) are collectively responsible for GPL transport to the OM (Fig. 1) (16). The asymmetric distribution of LPS and GPLs in the OM is important for OM integrity: should GPLs appear in the outer leaflet, the maintenance of lipid asymmetry (Mla) pathway exists to remove them and return them back into the CM (Fig. 1) (11, 17, 18).

The OM is rich in proteins. Transmembrane β-barrel proteins (termed OMPs) are highly abundant and perform myriad functions, including acting as generalized or specific pores for nutrient uptake, antibiotic efflux, and protein secretion (1). Unfolded OMPs are secreted across the CM by the SecYEG translocon and reach the OM via a network of redundant chaperones that ensure these hydrophobic proteins do not misfold in the aqueous periplasmic environment (19, 20). Chaperones deliver nascent OMPs to the β-barrel assembly machine (Bam), which will fold and insert the OMP into the OM bilayer (20–22). A second class of secreted proteins is also present in the OM, the lipoproteins (23, 24). Following their secretion, lipoproteins undergo additional modification in the CM to become triacylated at their N-terminal residue (25–27). Triacylation anchors lipoproteins into the CM bilayer. While some lipoproteins are intended to remain in the CM, the overwhelming majority are targeted for localization in the OM (28). The “localization of lipoproteins” (Lol) pathway sorts and traffics specific lipoproteins: OM-targeted lipoproteins are extracted from the CM, ferried across the periplasm, and finally anchored into the OM bilayer (24, 29).

Now that we have a broader view of the *E. coli* OM assembly pathways, we can see that the action of each pathway is restricted to acting on one distinct component of the OM. Obviously, a pre-existing OM is required for any of the OM assembly pathways to function, so there is also a fundamental interdependence. Yet, there is also a more discreet interdependence, where the biogenesis of one pathway relies on the function of another. For example, to build a new LptDE complex in the OM that will direct LPS transport into the outer leaflet, Bam is required to fold the LptD β-barrel and the Lol pathway is required to deliver the LptE lipoprotein. Bam itself relies on four OM lipoproteins, BamBCDE, with BamD being essential in wild-type *E. coli* (22, 30–32). In the GPL transport systems, the Mla pathway relies on the MlaA OM lipoprotein component and at least one of the AsmA pathways requires an OM lipoprotein (17, 33). The Lol lipoprotein trafficking pathway itself, somewhat paradoxically, requires the presence of the OM lipoprotein LolB (24, 29, 34).

Clearly, lipoprotein trafficking is a critical hub for building the OM—each of the biogenesis pathways must be supplied with one or more lipoprotein components needed for their function. This *E. coli*-centric view of OM assembly makes it impossible to imagine how an OM might be built without lipoproteins. Yet, diderm Terrabacteria apparently do exactly this—they build their OM with homologs of Bam, Lpt, AsmA, and Mla pathways but lack any OM lipoproteins whatsoever and, appropriately, lack a Lol pathway (5, 35). The contrast to *E. coli* is stark and begs the question: why has OM biogenesis in *E. coli* (and many Proteobacteria, Bacteroidetes, *et al.*) been so thoroughly rewired to fundamentally depend on OM lipoproteins?

## HOW TO LIPO- A PROTEIN, AND WHY THIS MIGHT BE A GREAT IDEA

Lipoproteins are produced by both monoderm and diderm bacteria (36). Only a subset of diderm species appears to also deliver lipoproteins to the OM (2). This fact supports the notion that the Lol trafficking pathway is the newest of the OM biogenesis pathways to have evolved, sometime after the ancient split between Terrabacteria and Gracilicutes. Apparently, the Lol pathway’s delivery of OM lipoproteins proved to be a marvelous evolutionary invention—OM assembly has been thoroughly rewired to almost fully depend on the action of essential OM lipoproteins in the Gracilicutes species studied. What made lipoproteins such a useful innovation and what advantages might lipoproteins offer over the other class of OM protein, β-barrel OMPs?

Lipoproteins possess a characteristic lipobox signal sequence that enables their secretion from the cytosol, typically, though not exclusively, via the SecYEG translocon (37–39). Once secreted, an invariant cysteine in the lipobox is *S*-diacylated by the enzyme Lgt to produce a prolipoprotein form which can subsequently be processed by signal peptidase II (LspA) to release the apolipoprotein from its signal peptide and leave the diacylated cysteine as the N-terminal residue (Cys^+1^) (25, 26, 40, 41). In diderms, the ordering of lipidation prior to protease processing is important—it ensures that nascent lipoproteins remain tethered to the CM by diacylation before they have been released from their signal peptide tether. Next, a final acylation is performed by the enzyme Lnt that *N*-acylates the Cys^+1^ amino group that is liberated by LspA (42, 43). Now, the mature, triacylated lipoprotein is finally formed. Lipoproteins are commonly triacylated in diderm bacteria and in some monoderms bacteria, but most monoderms lost Lnt and either simply produce diacylated lipoproteins or find more elaborate ways to additionally modify their lipoproteins (36, 44, 45).

In *E. coli*, triacylation is a prerequisite for subsequent trafficking to the OM via the Lol system (46–48). As you may expect, given that there are several essential OM lipoproteins, each of the maturation enzymes is accordingly essential for viability. Peptide-based rules allow the cell to sort synthesized lipoproteins into those that will be permanently retained in the CM from those (the majority) that will be trafficked to the OM; these peptide sorting sequences are well understood for only very few species, and they appear to vary widely (49–53).

Lipidation is a way of anchoring proteins to the longitudinal plane of a membrane (either the CM or the OM). However, integral transmembrane proteins are also fixed in this plane of the membrane, via either α-helical transmembrane domains in the CM or β-barrel transmembrane domains in the OM. So, what benefit is there to be a lipoprotein compared to being a protein attached to the membrane via a transmembrane domain? In principle, the signal sequence is itself an α-helical transmembrane domain, so why is it not sufficient for CM lipoproteins? The answer is not readily apparent, but there must be a discernable advantage to being a lipo-protein; monoderm bacteria clearly produce lipoproteins, preferring to lipid anchor such proteins in the CM rather than rely only a transmembrane domain. While lipoproteins are non-essential in many monoderm species and it is possible to delete the *lgt*, *lspA*, or *lnt*, genes, such deletion mutants exhibit poor growth, at least hinting at some underlying advantage of lipidation even in the CM (36, 54).

The OM is rich in lipoproteins that perform diverse functions even beyond helping build the OM: some are participants in cell wall synthesis and remodeling, others are key components of secretion systems, and others act as sensors of the cell envelope environment (55–65). In principle, all these OM lipoproteins could be alternately tethered to the OM via β-barrel domains. So, what is the advantage of being a lipo-protein? In the OM, the lipoprotein’s advantage might be clearer to discern. Recent measurements show that transmembrane β-barrel OMPs, exhibit extremely slow diffusion in the OM; indeed, they appear to cluster together into immobile OMP “islands” (66). OM lipoproteins, on the other hand, have been shown to exhibit rapid diffusion in the OM (67, 68). The ability to diffuse rapidly brings the benefit of being able to function repeatedly at multiple sites through the cell envelope. For example, redundantly essential OM lipoproteins (LpoA and LpoB) diffuse to coordinate cell wall growth and repair at sites where the peptidoglycan mesh is stretched or defective (69, 70). If such lipoproteins were alternately fixed to the OM via β-barrel anchoring, they would have to be synthesized and placed precisely at sites where they are currently needed or will be needed in the future.

The idea that diffusibility could be the major innovation offered by OM lipoproteins may be, at least indirectly, supported by the curious and repeated observations that many individual lipoproteins do not necessarily need to be lipo- at all! In many instances, engineering a replacement of the lipobox signal sequence with the signal sequence of soluble periplasmic protein yields soluble variants that are free to diffuse throughout the periplasm and remain functional, despite being no longer tethered to the OM (71–73). Why, then, are not lipoproteins just made as diffusible proteins in the periplasm—why the lipid tether? All the re-engineering experiments rely on multicopy expression plasmids and hence may mask the potential inherent benefits of being diffusible but also being restricted to the longitudinal plane of the OM. Furthermore, while re-engineering an individual lipoprotein in multicopy is possible with low fitness costs, wholesale changes of all ~100 *E*. *coli* OM lipoproteins into soluble periplasmic proteins are likely to seriously compound fitness costs.

In addition to diffusibility, there may be other benefits to having proteins tethered to the OM as lipoproteins. In the Bam pathway, the accessory BamBCDE lipoproteins appear to regulate BamA activity rather than actively participate in OMP folding (74–78). Had the globular periplasmic domains of BamBCDE been attached to the OM via β-barrel domains, this would mean that the biogenesis of BamA regulators would first require unregulated BamA activity to fold their β-barrel transmembrane domains. Moreover, at least in *E. coli*, it seems that BamA assembles and releases OMPs into a proximal, local environment, surrounding itself with an island of the OMPs it recently assembled (66). Had BamBCDE been tethered to the OM via β-barrel domains, each would occupy a considerable portion of the OMP island most proximal to BamA. Since they are produced as lipoproteins, BamBCDE neither require BamA for their biogenesis nor occupy space in the OMP island, both presumably favorable features for the Bam system. In addition, at least *in vitro*, lipoproteins BamB and BamD can independently assist nascent BamA to fold its own β-barrel into the bilayer (79). Since BamB and BamD OM localization is only reliant on the Lol system, they can define the site of a future OMP island and then help assemble the BamA proteins that will help to generate the OMP island.

While all diderm bacteria produce BamA, some lack any of the BamBCDE lipoproteins—how do they accomplish efficient OMP assembly with just BamA? First, there is already evidence of species-level diversity in Bam lipoprotein composition. For example, the Proteobacteria *C. crescentus* encodes a BamF lipoprotein that is a novel addition to this organism’s Bam machine, although BamF function in OMP biogenesis remains only loosely defined (80). It is possible that novel, distinct Bam OM lipoproteins may exist throughout distantly related diderm bacteria that have a Lol system to traffic them to the OM (e.g., *Fusobacterium nucleatum* which lacks any recognizable Bam lipoproteins but produces a Lol system and, at least, the OM lipoprotein MlaA) (81). Additionally, while much has been learned about the physical properties of the *E. coli* OM and how these curtail OMP diffusion, the properties of the OM among evolutionarily distant bacteria may differ. If OMP diffusibility is not highly restricted, there may not be the same need for lipoproteins. Yet, the δ-proteobacteria *Myxococcus xanthus* produce an OM with surprising fluidity—allowing even wholesale exchange of OM material between adjacent bacteria—and these bacteria nonetheless still produce OM lipoproteins. We currently know little about the biophysical properties of the OM among diverse diderms.

## N-ACYLATION: GREASING UP FOR A RIDE THROUGH THE LOL PATHWAY

The first two lipoprotein maturation enzymes (Lgt and LspA) are highly conserved throughout all bacteria and are essential in diderm species. The acyltransferase Lnt performs the third and final maturation reaction, acylation of a lipoprotein’s Cys^+1^ N-terminus. In *E. coli*, Lnt is essential, but its essentiality can be bypassed if LolCDE is overproduced (48). This observation implied that LolCDE has poor affinity for diacylated lipoproteins (which overproduction likely overcomes), leading to the idea that triacylation is a prerequisite for any lipoprotein to be trafficked to the OM via the Lol pathway in wild-type *E. coli*. A more recent study suggests that, rather than triacylation *per se*, *N*-acylation is in fact what licenses a lipoprotein to enter Lol trafficking pathway (45).

The Lit acyltransferase enzyme from Firmicutes *Enterococcus faecalis* and *Bacillus cereus N*-acylates lipoproteins, but rather than use a GPL acyl donor like Lnt, Lit simply takes an acyl chain from the lipoprotein’s *S*-diacylated Cys^+1^ residue (attached by Lgt) (45). Hence, Lit production yields diacylated lipoproteins with one *S*- and one *N*-acyl modification (45). LolCDE can apparently recognize such non-native lipoproteins quite efficiently*—lit* can complement *lnt* deletion to sustain *E. coli*. While native levels of *E. coli* LolCDE can recognize and traffic the unusually acylated lipoproteins, their trafficking is clearly not optimal (45).

Acylation status is apparently a critical requirement for entering LolCDE but Lol is generally indifferent to the peptide sequence of the lipoproteins it traffics. While having a flexible linker region between the Cys^+1^ residue and the globular domain of the lipoprotein aids in its efficient trafficking, Lol does not generally seem to discriminate on the sequence of a lipoprotein polypeptide. Indeed, appending a lipoprotein signal peptide to heterologous proteins (such as fluorescent proteins) can localize them in the OM via Lol (67, 82). The *E. coli* Lol paradigm only imposes a specific requirement for amino acids that are adjacent to the Cys^+1^ for a lipoprotein to avoid LolCDE and remain in the CM (83, 84). Hence, the *E. coli* paradigm invokes the idea that the default state of lipoproteins is to engage with LolCDE and end up in the OM—consistent with the fact that the majority of *E. coli* lipoproteins are indeed OM localized. Lipoprotein acylation status may be an important feature that helps LolCDE to discriminate between lipoproteins in the CM awaiting trafficking and the sea of GPLs that surround the transporter. The Lgt *S*-diacylation uses a diacylated GPL donor; hence, the GPL donor and the lipoprotein product have indistinguishable acylation. On the other hand, LolCDE could clearly discern a lipoprotein from a GPL by the presence of additional *N*-acylation.

Even among Proteobacteria, the essentiality of *N*-acylation is not a universal feature (85). While almost all Gracilicutes produce Lnt—and hence likely produce *N*-acylated lipoproteins—some species tolerate *lnt* deletion, at least during laboratory culturing. Examples include γ-Proteobacteria *Francisella tularensis* and *Acinetobacter* sp., as well as β-Proteobacteria *N. gonorrhea (85, 86*). Mutants lacking *lnt* produce a defective, leaky OM and exhibit impaired growth—there is certainly a fitness cost for lacking *N*-acylated lipoproteins (86). The trade-off for this fitness cost might be the ability to evade immune recognition. Distinct innate immunity sensors—the Toll-like receptors—sense either diacylated lipoproteins (TLR2-TLR1 heterodimers) or triacylated lipoproteins (TLR2-TLR6 heterodimers). This raises the question of whether some bacteria (and their Lol system) have the flexibility to selectively alter lipoproteins production between di- and tri-acylation during infection (87).

**Fig 2:**
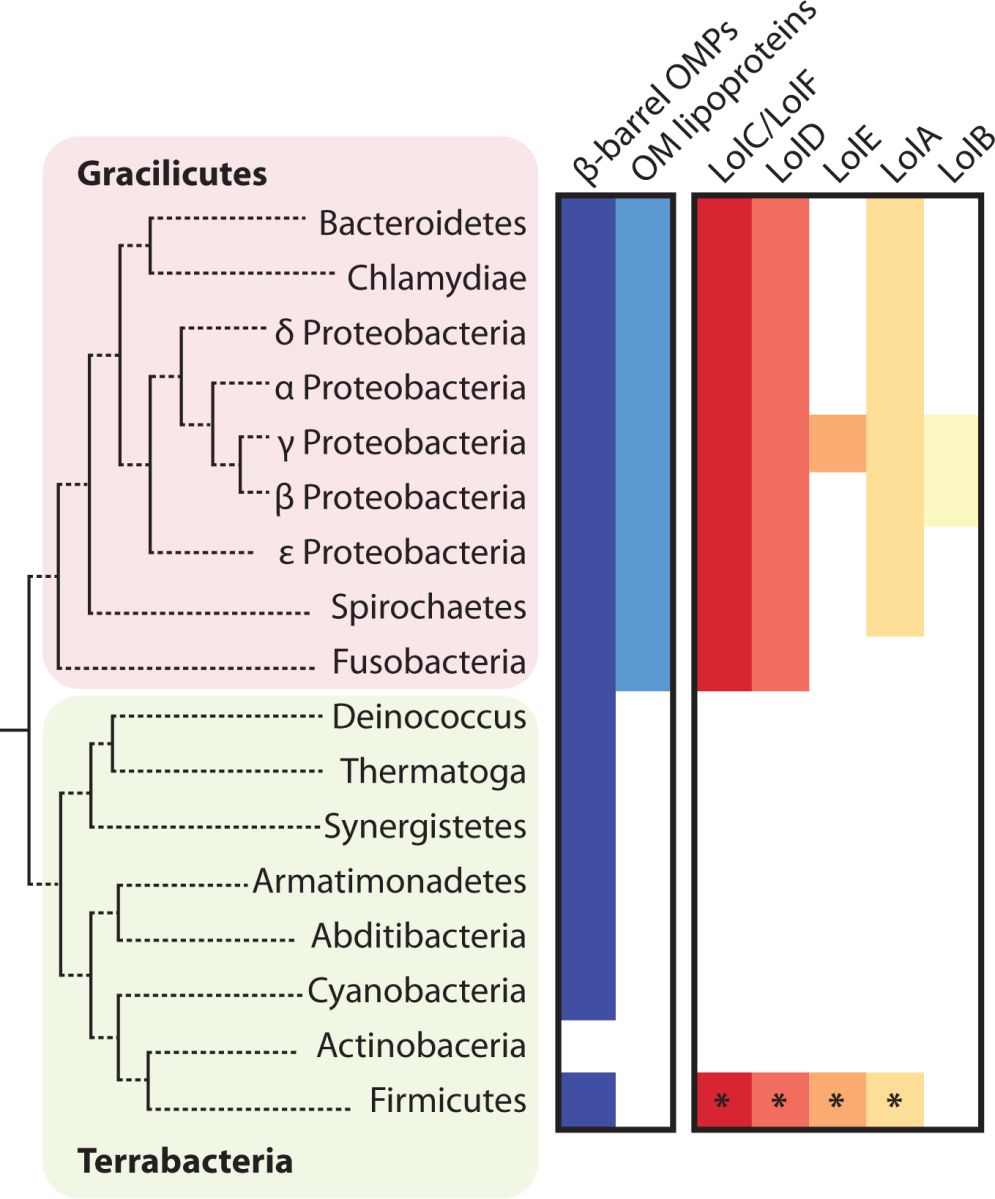
OM protein features and Lol pathway conservation among diderm species of selected phyla and classes. Colored squares indicate presence of one or more species with the feature, white squares indicate complete absence of species with the feature. The diderm architecture is ancient and β-barrel OMP assembly is highly conserved (defined as presence of BamA or OmpM homologs). OM lipoproteins are much less widely produced and are likely a newer addition to the cell envelope (defined as presence of either *bamBCDE*, *lptE*, *mlaA*, *lolB*, or *pal* homologous lipoprotein genes). The Lol pathway is present to traffic OM lipoproteins. The presence of *lolA* and an operon organization of *lolF-lolD* are typical. *lolB* is not identifiable outside γ- and β-Proteobacteria. The *E. coli lolC-lolD-lolE* operon organization is restricted to only a narrow subset of γ-Proteobacteria. The Lol pathway paradigm of *E. coli*, consisting of LolCDE, LolA, and LolB, is highly atypical among diderm bacteria, and even among Proteobacteria. Diderm Firmicutes in the *Halanaerobiaceae* family likely horizontally acquired *lolCDE* and *lolA* (indicated by asterisk).

The ability of a species to tolerate *lnt* deletion correlates with them producing a different form of the LolCDE transporter. In species where Lnt is essential, the LolCDE transporter consists of a heterodimeric LolC and LolE transmembrane complex that couples with the cytosolic LolD ATPase. Meanwhile, the species that tolerate the loss of *lnt* couple LolD to a homodimeric transmembrane complex of LolF, a protein that strongly resembles a chimera of LolC and LolE (85). The LolDF architecture is the most common among Gracilicutes and it may represent an ancestral system to LolCDE which is only present in a narrow subset of γ-Proteobacterial species that are closely related to *E. coli* (Fig. 2) (29). In fact, among Terrabacterial diderm, only *Halanabacteria* appear to have the Lol pathway, likely acquired by horizontal gene transfer from a γ-Proteobacterium since it appears to be the LolCDE form (2)

The relative advantages or disadvantages of the alternate LolDF or LolCDE architectures are still unclear. In the LolC and LolE system, discreet non-redundant functions are split between the two proteins: LolC is tasked with recruiting the LolA chaperone, while LolE is left to handle the extracted lipoprotein that will be LolA’s cargo (88–92). In the homodimeric LolF complex, presumably either component can do either of those functions, though it would seem sensible for the LolF proteins to coordinate in some way to avoid, say, both LolF proteins recruiting LolA and neither handling the lipoprotein cargo.

## TO LolB OR NOT TO LolB, THAT IS THE QUESTION

In *E. coli*, the final step of trafficking is performed by the OM-resident LolB insertase, itself a lipoprotein (34, 93, 94). LolB receives lipoprotein cargo from LolA and anchors its acyl chains into the OM bilayer. The functions of the LolA chaperone and the LolB insertase are separated in *E. coli* and neither protein can substitute for the other; deletion of either *lolA* or *lolB* is lethal in wild-type *E. coli*. A long-ago noted curiosity about the Lol system is that not all components of the system co-occur in all organisms despite their individual essentiality in the *E. coli* system (Fig. 2). There is a broad conservation of LolCDE/LolDF transporter genes and of the LolA chaperone gene throughout Gracilicutes (Proteobacteria, Bacteroidetes, Spirochaetes, and others) (Fig. 2) (29, 85). LolB, on the other hand, is only present in γ- and β-Proteobacteria (95). The pattern of *lolB* conservation implies that it emerged quite recently, sometime after γ- and β-Proteobacterial phyla split from α-Proteobacteria. LolB in other phyla, if present at all, must at least be highly sequence divergent from Proteobacteria, and hence currently unrecognizable.

The *E. coli* lipoprotein trafficking model dictates that in the many LolB-lacking bacteria we are left, bizarrely, with an apparently lethally incomplete Lol pathway. Yet, these LolB-lacking species clearly produce OM-localized lipoproteins (e.g., Bam lipoproteins) (Fig. 2). Obviously, lipoprotein trafficking must be completed in another manner. Since LolA is the final, recognizable Lol component in LolB-lacking bacteria, we suspect the pathway terminates with the action of LolA. We showed that at least one such LolA protein (from *Caulobacter vibrioides*, *nee C. crescentus*) behaved as a bifunctional chaperone and insertase—capable of substituting for both LolA and LolB when produced in *E. coli* (95). Indeed, the predicted *Caulobacter* LolA structure appeared to be chimeric, and engineering an analogous chimera of *E. coli* LolA-LolB yielded a similarly bifunctional chaperone-insertase (95). Hence, we now have a glimpse of how a bifunctional LolA homolog may complete the trafficking pathway solo in *Caulobacter*. The generality of this solution awaits additional testing.

We can rationalize the benefit of producing LolB, an OM lipoprotein, quite clearly—since it resides in the OM, it ensures all OM-targeted lipoprotein cargo ferried by LolA is delivered to the correct membrane. At least two *E. coli* lipoproteins, Lpp and OsmB, are lethally toxic if allowed to mislocalize in the CM (96, 97). The emergence of an OM anchored LolB, ensures mis-insertion from a bifunctional LolA back into the CM can never happen. This may have allowed for lipoproteins such as Lpp and OsmB to emerge. Indeed, the production of Lpp and OsmB lipoproteins is very narrowly restricted to *E. coli* and its very close, LolB-producing, cousins.

## LPT SYSTEMS LOOKING FOR WORK—DO THEY TRAFFIC LIPOPROTEINS?

A function for the Lpt system in lipoprotein trafficking was recently proposed from studies of the Spirochete *Borrelia burgdorferi (98*). Spirochetes produce an OM that lacks LPS. Instead, *B. burgdorferi*, *Treponema pallidum*, and other Spirochete species produce distinct glycolipids that are on the cell surface. *B. burgdorferi* is also suggested to substantially fill its OM outer leaflet with lipoproteins. Surface localization of distinct lipoproteins occurs in many species—even *E. coli* produces a few surface-exposed lipoproteins. However, *B. burgdorferi* is suggested to, uniquely, localize its lipoproteins at the cell’s surface by default, with only some lipoproteins adopting a periplasmic localization that is more typical of *E. coli* lipoproteins (99). The mechanism by which *B. burgdorferi* places its lipoproteins onto the cell surface has remained enigmatic. *B. burgdorferi* produces a LolDF-LolA trafficking system, lacking LolB. Earlier findings suggested that *B. burgdorferi* surface lipoproteins are first delivered to the OM and then a “flippase” protein in the OM translocates their globular domain across the OM bilayer (100). Indeed, depletion of BamA was found to prevent lipoprotein surface localization, raising the prospect that the elusive flippase might be a β-barrel OMP (101). Curiously, subsequent studies of *E. coli* now make it clear that BamA itself can aid lipoprotein surface localization—the OM lipoprotein RcsF is translocated onto the cell surface by BamA while it assembles a β-barrel OMP, resulting in RcsF adopting a complex trans-OM topology via the OMP lumen (58, 74, 75, 102).

A new study proposes that the *B. burgdorferi* flippase OMP is the LptD homolog (98). In protease shaving experiments—where Proteinase K is used to liberate surface-exposed peptides—cells depleted for LptD via CRISPRi were found to yield fewer protease-liberated peptides from surface lipoproteins, implying that these lipoproteins failed to properly translocate to the cell surface when LptD levels were limiting (98). While that study supported a role for LptD in lipoprotein surface localization, a later *B. burgdorferi* study—that also relied on protease shaving—came to the opposite conclusion, that LptD plays no role in surface lipoprotein biogenesis (103). Using a regulated *lptD* depletion construct, the later study found that lowered levels of LptD did not alter the release of peptides from a surface lipoprotein when Proteinase K was added (103). Since both studies relied on conceptually similar approaches (protease shaving) and both assessed the CspA surface lipoprotein and yet came to opposing conclusions, a role for LptD in surface lipoprotein localization in *B. burgdorferi* remains under debate.

Significant questions regarding Lpt-mediated lipoprotein trafficking remain to be explored: is the biogenesis of surface OM lipoproteins fully independent of LolDF and LolA in *B. burgdorferi*, do they solely traverse the Lpt pathway? At what point does discrimination between the two systems occur? Is this role for Lpt unique to *B. burgdorferi*, or does it extend further into Spirochetes and maybe even beyond? The hypothesis that the Lpt system may allow lipoprotein trafficking has several attractive features in an evolutionary context. The Lpt pathway pre-dates the Lol pathway in diderm bacteria (2). If lipoproteins once flowed to the OM along the Lpt transenvelope bridge (and may still do so, as was suggested for *B. burgdorferi*), this would provide a neat way for the LptD accessory lipoprotein LptE to emerge through capture (104). Similarly, ancestral diderms produced BamA without producing any identifiable accessory Bam lipoproteins (2, 5). If Lpt served as an ancient lipoprotein transport system, BamA may have acquired its lipoproteins as a consequence of its work folding the LptD β-barrel.

There are other observations that suggest retention of Lpt, even in the absence of LPS, may be functionally useful. Retention of the Lpt system even after the loss of LPS synthesis has repeatedly occurred among diderms—many diderm Terrabacterial species are examples, in addition to Spirochetes (2, 5). Diderm Terrabacteria and Spirochaetes have also retained the Mla pathway (albeit in modified forms) (105). The apparent pressure to retain both Lpt and the Mla system could imply an importance of lipid asymmetry in the OM bilayer; rather than maintaining the asymmetric distribution of GPLs and LPS as in *E. coli*, the asymmetry might be between GPLs and other glycolipid forms. The emerging model that proteins of the AsmA family deliver GPLs to the inner leaflet of the OM might only explain how only one side of an OM bilayer is formed. Cells would need a way to fill the outer leaflet too; in a broader sense, this is the task of the Lpt pathway in *E. coli*. Perhaps, in non-LPS-producing bacteria, the Lpt system has been adapted and retained to function analogously, filling the OM outer leaflet with whatever glycolipids (or lipids more generally) that the cell produces. It is currently unknown how glycolipids reach the OM or cell surface of Spirochetes. The Lpt system being responsible for Spirochete glycolipid transport would be a reasonable extension of its well-defined role in *E. coli*, but such a role currently remains entirely hypothetical. Finally, it is also worth considering that LPS-lacking diderm Terrabacteria have retained the Lpt system, yet apparently do not localize any of the lipoproteins they synthesize in their OM.

## CONCLUSIONS

Compared to the other OM biogenesis pathways, the relatively recent ascent of the Lol pathway allows us a curious glimpse of an OM biogenesis system adapting over time. It seems that the earliest version of the Lol pathway consisted of LolDF and LolA. This version of the Lol system is by far the most widely prevalent among distantly related Gracilicutes. The evolutionary origins of LolDF and LolA are not readily apparent. LolDF shares similarities with MacAB efflux pump components, so it may have adapted from ancestral efflux systems (92). Indeed, TolC, the OM pore that functions with efflux system like MacAB is highly conserved, even in diderm Terrabacteria (5, 106). The origin of the LolA periplasmic chaperone is completely unclear.

The next step in Lol pathway innovation was an expansion in the number of components: LolDF working with LolA and now also LolB. The origin of LolB may have been LolA itself since: (i) a LolA homolog from a species that lacks a dedicated LolB insertase appears to possess twin chaperone and insertase activities (95) and (ii) LolA and LolB share significant sequence and structural similarity (107). Either *lolA* gene duplication or horizontal acquisition of a second *lolA*, followed by segregation of the chaperone and insertase functions between the *lolA* paralogs may have yielded a two-protein LolA-LolB system. This mechanism for LolB’s ascent is still theoretical. As discussed, the appearance of LolB and the LolDF-LolA-LolB version of the Lol pathway was likely relatively recent since it only appears in γ- and β-Proteobacteria.

The third version of the Lol pathway, consisting of LolCDE-LolA-LolB is the one we are most familiar with, given that it was found and dissected in laboratory *E. coli* strains. However, this version of the Lol pathway is the least common and hence probably the newest. The LolCDE arrangement is only produced by a handful of γ-Proteobacterial such as the Enterobacterales and Pseudomonadales. Since LolF has amino acid features that are uniquely divided between LolC and LolE, it is tempting to speculate that LolF was the source for LolC and LolE (85). Indeed, genetic synteny between *lolF* and *lolD* might offer a clue for the origins of *lolC* and *lolE. lolF* and *lolD* are in an operon in all bacteria and the *lolF-lolD* organization is overwhelmingly common. However, a few γ-Proteobacteria (e.g., some species belonging to Alteromonadales and Pasteurellales, including *Haemophilus influenzae*) arrange the operon in a *lolD-lolF* structure and have a *lolC* homolog elsewhere in the genome. Perhaps the *lolC-lolD-lolE* operon arose following recombination with horizontally acquired *lolD-lolF* DNA which might have resulted in a *lolF-lolD-lolF* genetic arrangement. The presence of duplicate genes flanking *lolD* would increase the chances of lethal *lolD* excision and would favor rapid genetic variation to, perhaps, ultimately yield a *lolC-lolD-lolE* operon structure.

While it is tempting to see increasing complexity in the Lol system as reflecting increased sophistication or efficiency in lipoprotein trafficking, such a view is likely mistaken. Each version of the Lol pathway is simply sufficient to meet the demands of the repertoire of OM-targeted lipoproteins produced in each species. What selective forces favored each iterative version of the Lol pathway to arise remains a fascinating question.

Among current efforts to develop new antibiotics that kill such pathogens, there is a focus on targeting the essential OM assembly pathways, including OM lipoprotein biogenesis and trafficking via Lol (108–112). Arguably, the most mechanistically impressive inhibitor of lipoprotein biogenesis is the natural product globomycin, which mimics a lipoprotein substrate to inhibit LspA signal peptidase (113, 114). No LspA mutant variants that are resistant to globomycin have ever been reported. Indeed, structural analysis implies that mutations in LspA that might avoid globomycin binding would also prevent lipoprotein substrate recognition, leading to loss-of-function and lethality (113). That finding raises the exciting prospect that LspA inhibition may avoid future evolution of target site resistance. However, globomycin is plagued by poor penetration across the OM of Proteobacteria (112). Recent globomycin analogs have improved LpsA targeting and yielded promising leads (115–117). The lipoprotein biogenesis processes that are accomplished by integral CM proteins Lgt, LspA, and Lnt are challenging to adapt for high-throughput *in vitro* inhibitor screening, but recent fluorescence-based click chemistry assays are leading the way forward (118, 119). Peptide-based inhibitors of the Lgt enzyme were recently identified in both *E. coli* and *Acinetobacter baumannii (120–122*). On the trafficking side, two LolFD/LolCDE inhibitors hold exciting promise, having been shown to effectively control infections in mouse models. A machine learning approach identified a potent *A. baumannii* LolF inhibitor (abaucin) with narrow spectrum against this pathogen (111). Similarly, an optimized form of previously characterized *E. coli* LolCDE inhibitors yielded lolamicin which targets pathogenic *E. coli* with exquisite selectivity (108). Relative to the other, more ancient OM assembly pathways, the Lol pathway varies considerably among diderms that cause high morbidity and mortality in humans. A useful feature of the Lol pathway’s recent evolution and relatively high diversity across species may be the ability to discover or develop highly targeted therapeutics that can selectively kill only a narrow set of pathogenic diderm species while sparing the many beneficial diderms that humans cultivate as part of their normal microbial flora (108, 111).
